# RETRACTION: Effect of Prophylactic Use of Cefazolin in Caesarean Section on Postoperative Infection: A Meta‐Analysis

**DOI:** 10.1111/iwj.70400

**Published:** 2025-03-21

## Abstract

**RETRACTION:** LvX. , RenX. , XuJ. , and WuH. , “Effect of Prophylactic Use of Cefazolin in Caesarean Section on Postoperative Infection: A Meta‐Analysis,” International Wound Journal 21, no. 4 (2024): e14740, 10.1111/iwj.14740.38522482 PMC10961181

The above article, published online on 24 March 2024, in Wiley Online Library (http://onlinelibrary.wiley.com/), has been retracted by agreement between the journal Editor in Chief, Professor Keith Harding; and John Wiley & Sons Ltd. Following an investigation by the publisher, all parties have concluded that this article was accepted solely on the basis of a compromised peer review process. The editors have therefore decided to retract the article. The authors did not respond to our notice regarding the retraction.

**RETRACTION:** X. Lv , X. Ren , J. Xu , and H. Wu , “Effect of Prophylactic Use of Cefazolin in Caesarean Section on Postoperative Infection: A Meta‐Analysis,” International Wound Journal 21, no. 4 (2024): e14740, 10.1111/iwj.14740.38522482 PMC10961181

The above article, published online on 24 March 2024, in Wiley Online Library (http://onlinelibrary.wiley.com/), has been retracted by agreement between the journal Editor in Chief, Professor Keith Harding; and John Wiley & Sons Ltd. Following an investigation by the publisher, all parties have concluded that this article was accepted solely on the basis of a compromised peer review process. The editors have therefore decided to retract the article. The authors did not respond to our notice regarding the retraction.

